# Association study between hypothalamic functional connectivity, early nutrition, and glucose levels in healthy children aged 6 years: The COGNIS study follow-up

**DOI:** 10.3389/fnut.2022.935740

**Published:** 2022-10-12

**Authors:** Estefanía Diéguez, Ana Nieto-Ruiz, Cristina Martín-Pérez, Natalia Sepúlveda-Valbuena, Florian Herrmann, Jesús Jiménez, Roser De-Castellar, Andrés Catena, José Antonio García-Santos, Mercedes G. Bermúdez, Cristina Campoy

**Affiliations:** ^1^Department of Paediatrics, School of Medicine, University of Granada, Granada, Spain; ^2^Instituto de Investigación Biosanitaria (ibs.GRANADA), Health Sciences Technological Park, Granada, Spain; ^3^EURISTIKOS Excellence Centre for Paediatric Research, Biomedical Research Centre, University of Granada, Granada, Spain; ^4^Psychology Department, Faculty of Education, University of Valladolid, Segovia, Spain; ^5^Nutrition and Biochemistry Department, Faculty of Sciences, Pontificia Universidad Javeriana, Bogotá, Colombia; ^6^Ordesa Laboratories, S.L., Sant Boi de Llobregat, Spain; ^7^Department of Experimental Psychology, School of Psychology, University of Granada, Granada, Spain; ^8^National Network of Research in Epidemiology and Public Health (CIBERESP), Institute of Health Carlos III (Granada's Node), Madrid, Spain

**Keywords:** neuroimaging, hypothalamus, mean glucose levels, milk fat globule membrane (MFGM), long chain polyunsaturated fatty acids (LC-PUFAs), synbiotics, eating behavior

## Abstract

Breastfeeding (BF) is the gold standard in infant nutrition; knowing how it influences brain connectivity would help understand the mechanisms involved, which would help close the nutritional gap between infant formulas and breast milk. We analyzed potential long-term differences depending on the diet with an experimental infant formula (EF), compared to a standard infant formula (SF) or breastfeeding (BF) during the first 18 months of life on children's hypothalamic functional connectivity (FC) assessed at 6 years old. A total of 62 children participating in the COGNIS randomized clinical trial (Clinical Trial Registration: www.ClinicalTrials.gov, identifier: NCT02094547) were included in this study. They were randomized to receive an SF (*n* = 22) or a bioactive nutrient-enriched EF (*n* = 20). BF children were also included as a control study group (BF: *n* = 20). Brain function was evaluated using functional magnetic resonance imaging (fMRI) and mean glucose levels were collected through a 24-h continuous glucose monitoring (CGM) device at 6 years old. Furthermore, nutrient intake was also analyzed during the first 18 months of life and at 6 years old through 3-day dietary intake records. Groups fed with EF and BF showed lower FC between the medial hypothalamus (MH) and the anterior cingulate cortex (ACC) in comparison with SF-fed children. Moreover, the BF children group showed lower FC between the MH and the left putamen extending to the middle insula, and higher FC between the MH and the inferior frontal gyrus (IFG) compared to the EF-fed children group. These areas are key regions within the salience network, which is involved in processing salience stimuli, eating motivation, and hedonic-driven desire to consume food. Indeed, current higher connectivity found on the MH-IFG network in the BF group was associated with lower simple sugars acceptable macronutrient distribution ranges (AMDRs) at 6 months of age. Regarding linoleic acid intake at 12 months old, a negative association with this network (MH-IFG) only in the BF group was found. In addition, BF children showed lower mean glucose levels compared to SF-fed children at 6 years old. Our results may point out a possible relationship between diet during the first 18 months of life and inclined proclivity for hedonic eating later in life.

**Clinical trial registration:**

https://www.clinicaltrials.gov/, identifier NCT02094547.

## Introduction

The first 1,000 days of life, from conception to 2 years of age, are crucial in brain development, and nutrition plays a key role. In fact, nutrition influences brain development not only after birth but also during the prenatal period as well (1). Thus, infancy is a critical window of opportunity and constitutes the period of greater plasticity throughout human brain development (2). Several environmental and genetic factors, not all of them easy to control, influence brain development; however, among them, nutrition is easily amendable (3, 4). Indeed, nutrition during early life is an important and modifiable factor responsible for shaping cognitive outcomes later in life. Although all nutrients are important for structural and functional brain development, energy, carbohydrates (CHs), proteins, and lipids are of particular importance (4, 5). Long-chain polyunsaturated fatty acids (LC-PUFAs) have been identified as essential for neurogenesis and synaptogenesis, affecting both prenatal and postnatal brain development (6–8).

It is known that there are notable differences in neurodevelopment and cognitive function between breastfed and formula-fed infants. Breast milk is the gold standard for infant feeding due to its composition in bioactive nutrients, and it is associated with short- and long-term health benefits (9, 10), as well as an optimal brain development (4, 11, 12). However, BF may not always be possible or suitable. Current infant formulas are being supplemented with bioactive ingredients to closely mimic the nutritional composition of breast milk and to obtain similar potential beneficial effects on infant growth and cognitive development (13). Several studies reported that supplementation of infant formulas with LC-PUFAs, such as arachidonic acid (ARA), eicosapentaenoic acid (EPA), docosahexaenoic acid (DHA) (10), specific oligosaccharides (14), and probiotics and prebiotics (15, 16), determines beneficial effects regarding neurodevelopment and cognitive function (4, 13, 17). In contrast, research efforts have shown that infant formulas supplemented with milk-fat globule membrane (MFGM) have positive results in neurological development as well (18, 19). Despite the studies mentioned above, there is still scarce evidence about how early nutrition affects cognitive function. Thus, in this study, we focused on a possible relationship between early diet and brain connectivity.

Increasing evidence suggests that the central nervous system plays a key role in glucose regulation and consequent development of metabolic disorders when altered, including type 2 diabetes (T2D) and obesity (20–23). Neuroimaging studies with functional magnetic resonance imaging (fMRI) have shown evidence in favor of brain involvement in glucose metabolism and regulation and how glucose dysregulation could affect brain functionality (24). The hypothalamus is one of the most important brain regions involved in the central control of feeding and energy expenditure (25). Indeed, it includes hedonic control of appetite by cortical and subcortical brain areas, processing external sensory information and reward (26). It is constituted by various nuclei [ventromedial nucleus of the hypothalamus (VMN), dorsomedial hypothalamic nucleus (DMH), lateral hypothalamus (LH), arcuate nucleus of the hypothalamus (ARC), and paraventricular nucleus (PVN)] containing specific glucose-sensing neurons that contribute to the regulation of glucose metabolism (27). The VMN contains many neural populations with different functions, such as promoting glucose utilization, restraining hepatic glucose production during normal blood glucose levels, and stimulating peripheral glucose uptake (28, 29). The DMH is implicated in the regulation of energy homeostasis, playing an important role in food intake and body weight (30). The LH has an essential role in the control of feeding behavior and metabolism, and it is also important in reward and reinforcement processes (31, 32). Finally, the ARC regulates energy intake, glucose metabolism, and energy expenditure (25).

The primary objective of this study was to analyze potential long-term differences depending on the diet with an experimental infant formula (EF), compared to a standard infant formula (SF) or breastfeeding (BF) during the first 18 months of life on children's hypothalamic network functional connectivity (FC) assessed at 6 years old. The secondary objective of this study was to study a potential association between nutrient intakes at 6, 12, and 18 months, and FC of the hypothalamus, including a possible correlation between mean glucose levels and hypothalamic FC in children at 6 years old.

## Materials and methods

### Ethics, informed consent, and permissions

The COGNIS study has been performed by following the updated Declaration of Helsinki II Principles (33, 34). Both projects and protocols were approved by the Research Bioethical Committee from the University of Granada (Spain), as well as the Bioethical Committees for Clinical Research from San Cecilio University Clinical and University Mother-Infant Hospitals of Granada (Spain). All families were informed about the procedures and each parent or legal guardian signed a written informed consent before involving their child in the study.

### Study design and subjects

The COGNIS study is a prospective, double-blind randomized clinical trial with a nutritional intervention (Clinical Trial Registration: www.ClinicalTrials.gov, identifier: NCT02094547). Detailed information about this study, including subject recruitment, design, and population characteristics, has been described elsewhere (35–37). Concisely, a total of 220 healthy Spanish infants were enrolled in the COGNIS study; of them, 170 were randomized (ratio of 1:1) to receive, during their first 18 months of life, either an SF (*n* = 85) or an EF (*n* = 85) enriched with milk-fat globule membrane (MFGM) components [10% of total protein content (wt:wt)], LC-PUFAs (DHA and ARA), synbiotics (fructooligosaccharides: inulin proportion of 1:1; Bifidobacterium longum subsp. infantis CECT7210 (Bifidobacterium infantis IM1) and Lactobacillus rhamnosus LCS-742], gangliosides, nucleotides, and sialic acid. The detailed nutritional composition of SF and EF can be found in Supplementary Table 2, and it has been described elsewhere (36, 38). In addition, 50 exclusively breastfed infants were included as a control group (BF: *n* = 50).

In addition, parents' baseline information was obtained at study entry, including age, preconceptional maternal body mass index (pBMI), gestational weight gain (GWG), smoking during pregnancy, educational level, place of residence, as well as employment and socioeconomic status. Postpartum depression was assessed with the Spanish version of the Edinburgh Postnatal Depression Scale (39), and parents' intelligence quotient (IQ) was evaluated using the G factor of the Cattell Intelligence test (40, 41).

**Figure 1:**
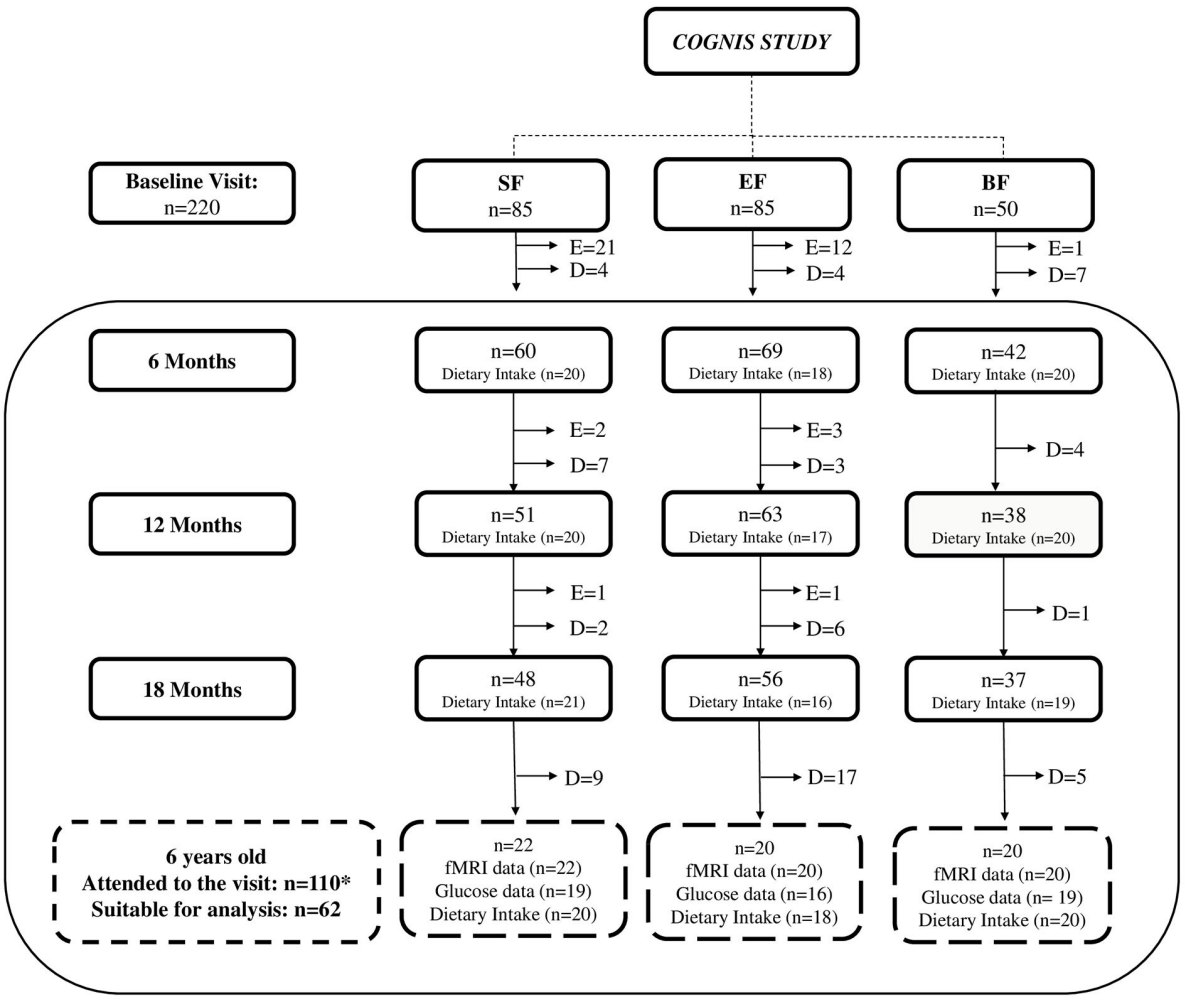
Participant flowchart from baseline visit to 6 years old. BF, breastfeeding; D, dropouts; E, exclusions; EF, experimental infant formula; fMRI, functional magnetic resonance imaging; *n*, sample size; SF, standard infant formula. Up to 18 months of life, a total of 40 infants were excluded in the groups fed with SF and EF as follows: 24 were excluded in the SF-fed group (1 due to perinatal hypoxia, 1 had growth deficiency, not related to the infant formula, 15 did not take the infant formula, 2 had colic, 3 were excluded due to lactose intolerance, 1 was excluded due to digestive surgical intervention, and 1 suffered from hydrocephalus); 16 were excluded in the EF-fed group (2 presented growth deficiency, not related to the infant formula, 2 had lactose intolerance, 11 did not take the infant formula, and 1 was excluded due to epileptic seizure). While in the BF group, one infant was excluded, because he was not exclusively breastfed beyond the 2 months of age, when he started to take only infant formula. In the follow-up visits, drop-outs were due to participants' parents deciding to withdraw from the study. After drop-outs, *110 children (SF: 39; EF: 39; BF: 32) attended the follow-up visit at 6 years old. Nonetheless, not all parents attending to the follow-up visit wanted their children to participate in the fMRI session or wear the 24-h continuous glucose monitoring device. Moreover, according to methodology previously described (42), some fMRI data were eliminated because image quality was not adequate due to excessive movement inside the scanner (SF: 15; EF: 19; BF: 4). In addition, mean glucose data were collected with a continuous glucose monitoring device for an average of 7 days. Those glucose data registered for <3 days were not included in final analysis (SF: 5; EF: 8; BF: 4). Finally, 62 children were included at 6 years old in the current analysis (SF: 22; EF: 20; BF: 20).

Dietary intake was evaluated from 6 to 18 months of age and at 6 years old through 3-day dietary intake records. After drop-outs, 110 (SF: 39; EF: 39; BF: 32) children attended a follow-up visit at 6 years old. Participants were asked to participate in an fMRI session, and mean glucose data were also collected for an average of 7 days by using a 24-h continuous glucose monitoring (CGM) device. It is worth noting that all children were healthy and within the normal range for brain function and glucose levels. Nonetheless, not all parents attending the follow-up visit wanted their children to participate in the fMRI session or wear the 24-h CGM device, and not all images from the fMRI session were suitable for analysis due to excessive movement inside the scanner (42). Thus, the current analysis included 62 children at 6 years old (SF: *n* = 22; EF: *n* = 20; BF: *n* = 20). A detailed participant flowchart from the baseline visit to 6 years old is shown in Figure 1.

### Functional resting-state magnetic resonance imaging (fMRI) procedure

#### Imaging data acquisition

Prior to the neuroimaging session, children were familiarized with the scanner's sounds and fMRI environment using a mock fMRI scanner. Brain data were acquired using a 3T magnetic resonance imaging (MRI) scanner equipped with a 32-channel phased-array head coil for reception (Magnetom Trio Siemens Medical System, Erlangen, Germany), located at the Mind, Brain, and Behavior Research Center (CIMCYC, University of Granada, Spain).

Functional resting-state imaging was obtained with a T2^*^-weighted echo-planar imaging (EPI) sequence using the following parameters: repetition time (TR) = 2,000 ms, echo time (TE) = 25 ms, flip angle = 80°, field of view (FOV) = 240 × 240 mm, matrix size = 68 × 68, number of slices = 35, and voxel size = 3.5 × 3.5 × 3.5 mm. We acquired 160 volumes for a total acquisition time of 5 min 20 s. Participants were instructed to keep calm and close their eyes during acquisition. A high-resolution T1-weighted anatomical image for each subject with 160 slices (TR = 8.3 ms; TE = 3.8 ms; flip angle = 8°; FOV = 240 × 240 mm; in-plane resolution = 0.94 × 0.94 × 1; slice thickness = 1 mm) was also acquired to discard gross radiological alterations and for preprocessing purposes.

#### Preprocessing of functional imaging data

Functional images were preprocessed using the CONNv17 FC toolbox (43), implemented in MatlabR2017a (The MathWorks Inc., Natick, Massachusetts, USA). The preprocessing pipeline included realignment, denoising of motion artifacts, and head motion (aCompCor (44), segmentation, coregistration to each participant's anatomical scan, normalization to an age-specific T1 template for pediatric studies (45), re-sliced to a 2-mm isotropic resolution in MNI space, and smoothing using a Gaussian kernel of 6 mm full-width at half-maximum [FWHM]). Additional steps after denoising included band-pass filtering of the BOLD time series (between 0.008 and 0.09 Hz) and linear detrending. After the motion artifacts and head motion detection, 34 children were excluded for excessive motion (which is to say, those with <4 min of data were excluded) (42).

### Dietary intake

Three-day dietary intake records were used to collect information about participants' dietary intake from 6 to 18 months of age and at 6 years old, based on the Food and Agriculture Organization of the United Nations (FAO) methods (46). In addition, the DIAL software (Alce Ingeniería, Madrid, Spain) (47) was used to convert food consumption data into macro- and micronutrient intakes. Nutrient intake was analyzed according to the dietary reference intakes (DRIs) (48), a set of reference values used to assess nutrient intake, to know whether the dietary intake is deficient (below recommendation), adequate (met recommendation), or excessive (exceeded recommendation), taking into account age (refer to Supplementary Methodology). Acceptable macronutrient distribution ranges (AMDRs) (48) were also calculated and classified as deficient (below recommendation), adequate (met recommendation), or excessive (exceeded recommendation) according to age (refer to Supplementary Methodology). AMDR stands for the energy that each macronutrient (CHs, proteins, or lipids) supplies to the total energy intake per day expressed as percentages.

### Continuous glucose monitoring (CGM)

To evaluate glucose homeostasis, a 24-h CGM was performed in children aged 6 years for an average of 7 days. Glucose levels were measured with the FreeStyle Glucose FlashMonitoring System (49) (http://www.freestylelibre.es; Reference 0086, Abbott Laboratories), which measures glucose levels in the interstitial fluid of subjects aged 4 years or older. Parents were instructed on how to use the device by trained personnel, and they told to scan the sensor before and after every meal and again 2 h after eating. Glucose data were downloaded using the FreeStyle LibreLink software (version 2.4.1; Abbott Laboratories) and, subsequently, analyzed to obtain mean glucose levels for each child every 24 h for at least 1 week.

### Statistical analysis

**General statistical analyses**. All statistical analyses were performed using IBM^®^ SPSS Statistics^®^ program, version 25.0 (SPSS Inc., Chicago, USA). Normally distributed variables were presented as mean and standard deviation (SD), and non-normal variables were presented as median and interquartile range (IQR). Categorical variables are displayed as frequencies and percentages. The ANOVA test for normally distributed variables, the Kruskal–Wallis test for non-normal continuous variables, and the chi-square or Fisher's test for categorical variables were performed. Bonferroni-corrected *post-hoc* comparisons were used to identify significant pair-wise group differences (corrected *p*-values < 0.05).

**Hypothalamic seed-based functional connectivity analyses**. As previously reported (50), respective seeds of interest were placed in the LH (x = +6, y = −10, z = −10) and the MH (x = +4, y = −2, z = −12), using 2-mm radius spheres distinguished per hemisphere. The MH included the ARC and VMN and parts of the DMH. In contrast, the LH seed was in the most posterior part of the hypothalamus to minimize overlap with the MH. First-level t-test maps were estimated for each of the LH and MH seeds by including its mean activity time courses [extracted using marsbar toolbox (51)] together with nuisance signals as predictors of interest and no interest in whole-brain linear regression analyses. Then, the left and right sides of each seed (LH and MH) were joined in a unique seed per region. Contrast images were generated for each subject by estimating the regression coefficient between all brain voxels and each seed's time series. Then, the contrast images were included in separate second-level t-test models to analyze study group differences together with variables of no interest, such as smoking during pregnancy and the type of delivery, and the sex and body mass index (BMI) of the children.

**Imaging thresholding criteria**. Minimum thresholds for the imaging analyses were calculated for all statistical comparisons by 1,000 Monte Carlo simulations using the cluster-extent-based AlphaSim thresholding approach (52) as implemented in the SPM RESTplusV1.2 toolbox. We included as input parameters an individual voxel threshold probability of 0.001, a cluster connection radius of 5 mm, and the actual smoothness of imaging data after model estimation, incorporating a whole-brain image mask.

**Correlation analyses**. Finally, partial correlations were carried out in order to explore the potential associations between brain connectivity and mean glucose levels measured at 6 years old, as well as between brain connectivity at 6 years of age and nutrient intake analyzed at 6, 12, and 18 months of age. These correlations were adjusted by the following confounder variables involved in brain development, including sex, maternal age, smoking during pregnancy, and socioeconomic status. All these variables have been named important factors regarding brain development in children (53–55). *P-*value of < 0.05 was considered statistically significant. The JASP software was used to carry out the aforementioned correlation analyses.

## Results

### Characteristics of the COGNIS study participants at 6 years old

The background and baseline characteristics of parents and children participating in this study are shown in Table 1. Differences were found in variables analyzed between the three study groups. Indeed, mothers and fathers from the BF group were significantly older than parents from the EF-fed group (p = 0.043; p = 0.014, respectively). Regarding socioeconomic status, parents from the BF group had a significantly higher socioeconomic status compared to both infant formula-fed groups (p = 0.009). Finally, due to the COGNIS study design, days of BF during the first 18 months of life significantly differed between BF and both formula-fed groups (p < 0.001). In contrast, parents' educational level and IQ were similar across the three study groups. No significant differences were detected regarding maternal pBMI, as well as GWG. Mothers were usually non-smokers during pregnancy, and all infants participating in the COGNIS study were born more frequently by vaginal delivery.

**Table 1:** Children's and parents' baseline characteristics at 6 years old depending on their type of diet during the first 18 months of life^1^.

		**SF (n = 22)**	**EF (n = 20)**	**BF (n = 20)**	**p^2^**
**Mother**					
Age (years)		30.77 ± 6.99^a, b^	30.40 ± 3.90^a^	33.45 ± 3.75^b^	**0.043**
pBMI (kg/m^2^)		23.98 ± 4.01	24.48 ± 3.29	24.48 ± 3.26	0.871
GWG (kg)		7.00 (7.50)	5.00 (6.25)	5.50 (4.75)	0.994
Type of delivery	Vaginal	16.00 (72.70%)	13.00 (65.00%)	15.00 (75.00%)	0.765
	Cesarean section	6.00 (27.30%)	7.00 (35.00%)	5.00 (25.00%)	
IQ (points)		105.23 ± 13.02	101.05 ± 16.40	108.55 ± 13.82	0.266
Educational level	NS/primary	2.00 (9.10%)	5.00 (25.00%)	1.00 (5.00%)	0.073
	Secondary	7.00 (31.80%)	5.00 (25.00%)	1.00 (5.00%)	
	VT	6.00 (27.30%)	7.00 (35.00%)	8.00 (40.00%)	
	University/PhD	7.00 (31.80%)	3.00 (15.00%)	10.00 (50.00%)	
Smoking during pregnancy	No	19.00 (86.40%)	18.00 (90.00%)	19.00 (95.00%)	0.864
	Yes	3.00 (13.60%)	2.00 (10.00%)	1.00 (5.00%)	
Postpartum depression	No	16.00 (72.70%)	17.00 (85.00%)	17.00 (85.00%)	0.557
	Yes	6.00 (27.30%)	3.00 (15.00%)	3.00 (15.00%)	
Employment status	Unemployed	5.00 (22.70%)	2.00 (10.00%)	3.00 (15.00%)	0.851
	Domestic work	1.00 (4.50%)	2.00 (10.00%)	1.00 (5.00%)	
	TC	1.00 (4.50%)	3.00 (15.00%)	2.00 (10.00%)	
	SE	15.00 (68.20%)	13.00 (65.00%)	14.00 (70.00%)	
Siblings	0	5.00 (22.70%)	3.00 (15.00%)	3.00 (15.00%)	0.770
	≥1	17.00 (77.30%)	17.00 (85.00%)	17.00 (85.00%)	
**Father**					
Age (years)		31.50 ± 7.25^a, b^	32.06 ± 4.63^a^	35.56 ± 3.05^b^	**0.014**
IQ (points)		108.86 ± 12.94	107.50 ± 14.91	106.78 ± 12.41	0.882
Educational level	NS/primary	4.00 (18.20%)	7.00 (35.00%)	4.00 (20.00%)	0.197
	Secondary	10.00 (45.50%)	6.00 (30.00%)	5.00 (25.00%)	
	VT	3.00 (13.60%)	6.00 (30.00%)	4.00 (20.00%)	
	University/PhD	5.00 (22.70%)	1.00 (5.00%)	7.00 (35.00%)	
Employment status	Unemployed	4.00 (18.20%)	0 (0.0%)	1.00 (5.00%)	0.279
	Domestic work	0 (0.0%)	0 (0.0%)	0 (0.0%)	
	TC	3.00 (13.60%)	1.00 (5.60%)	2.00 (10.00%)	
	SE	15.00 (68.20%)	17.00 (94.40%)	17.00 (85.00%)	
**Parents**					
Socioeconomic status	Low	5.00 (22.70%)	3.00 (15.00%)	0 (0.0%)	**0.009**
	Middle-low	10.00 (45.50%)	9.00 (45.00%)	5.00 (25.00%)	
	Middle-high	6.00 (27.30%)	8.00 (40.00%)	8.00 (40.00%)	
	High	1.00 (4.50%)^a^	0 (0.0%)^a^	7.00 (35.00%)^b^	
Place of residence	Urban	11.00 (50.00%)	6.00 (30.00%)	3.00 (15.00%)	0.051
	Rural	11.00 (50.00%)	14.00 (70.00%)	17.00 (85.00%)	
**Neonate**					
Gestational age (weeks)		40.00 (2.00)	40.00 (2.00)	39.00 (3.00)	0.593
Breastfeeding (days)		11.50 (35.25)^a^	17.50 (28.00)^a^	480.00 (270.00)^b^	**< 0.001**
Sex of the child	Boy	15.00 (68.20%)	13.00 (65.00%)	10.00 (50.00%)	0.442
	Girl	7.00 (31.80%)	7.00 (35.00%)	10.00 (50.00%)	
**Children at 6 years old**					
BMI (kg/m^2^)		15.92 (1.63)	16.26 (2.36)	15.70 (2.40)	0.819
HC (cm)		51.28 ± 1.62	51.74 ± 1.90	51.48 ± 0.91	0.716
Mean glucose (mg/dl)		101.24 (15.34)^a^	95.73 (15.82)^a, b^	91.16 (13.17)^b^	**0.027**
Macronutrients' intake (g/day)	Energy (kcal/day)	1741.91 ± 112.46	1811.19 ± 193.94	1711.48 ± 108.47	0.100
	Total carbohydrates	177.00 (36.00)	191.50 (48.00)	173.50 (34.50)	0.522
	Simple sugars	91.70 ± 14.61	81.63 ± 17.62	92.28 ± 16.19	0.086
	Total lipids	74.63 ± 20.97	76.38 ± 16.33	72.26 ± 9.71	0.638
	Linoleic acid	5.80 (4.00)	6.60 (5.00)	6.95 (4.20)	0.758
	Total proteins	61.50 (23.15)	68.80 (18.70)	64.90 (13.02)	0.442

1 Data are presented as mean ± stardard deviation (SD) for parametrically distributed data, n (%) for categorical data, and median (interquartile ranges) for non parametrically distributed data.

2 P-values for overall differences between COGNIS groups. ANOVA for normally distributed variables, Kruskal–Wallis test for non-normal continuous variables, and chi-square or Fisher's test for categorical variables. Values not sharing the same suffix (ab) were significantly different in the Bonferroni *post-hoc* test. P-values of < 0.05 are highlighted in bold. BF, breastfed infants; BMI, body mass index; EF, experimental infant formula; GWG, gestational weight gain; HC, head circumference; IQ, intelligence quotient; kcal, kilocalories; NS, no schooling; pBMI, preconceptional body mass index; SE, stable employment; SF, standard infant formula; TC, Temporary contract; VT, Vocational training.

Furthermore, at 6 years old, children did not differ in their BMI and head circumference (HC). Although children aged 6 years participating in the COGNIS study presented mean glucose values between 70 and 125 mg/dl (normal range after at least 8 h of fasting) (56), BF children showed lower mean glucose levels compared to the SF-fed group (p = 0.027), and there were no differences between children fed with BF and EF. Finally, no differences were found between study groups regarding the intake of macronutrients at 6 years of age.

### Differences between COGNIS groups in resting-state functional connectivity of the hypothalamus at 6 years old

**Figure 2:**
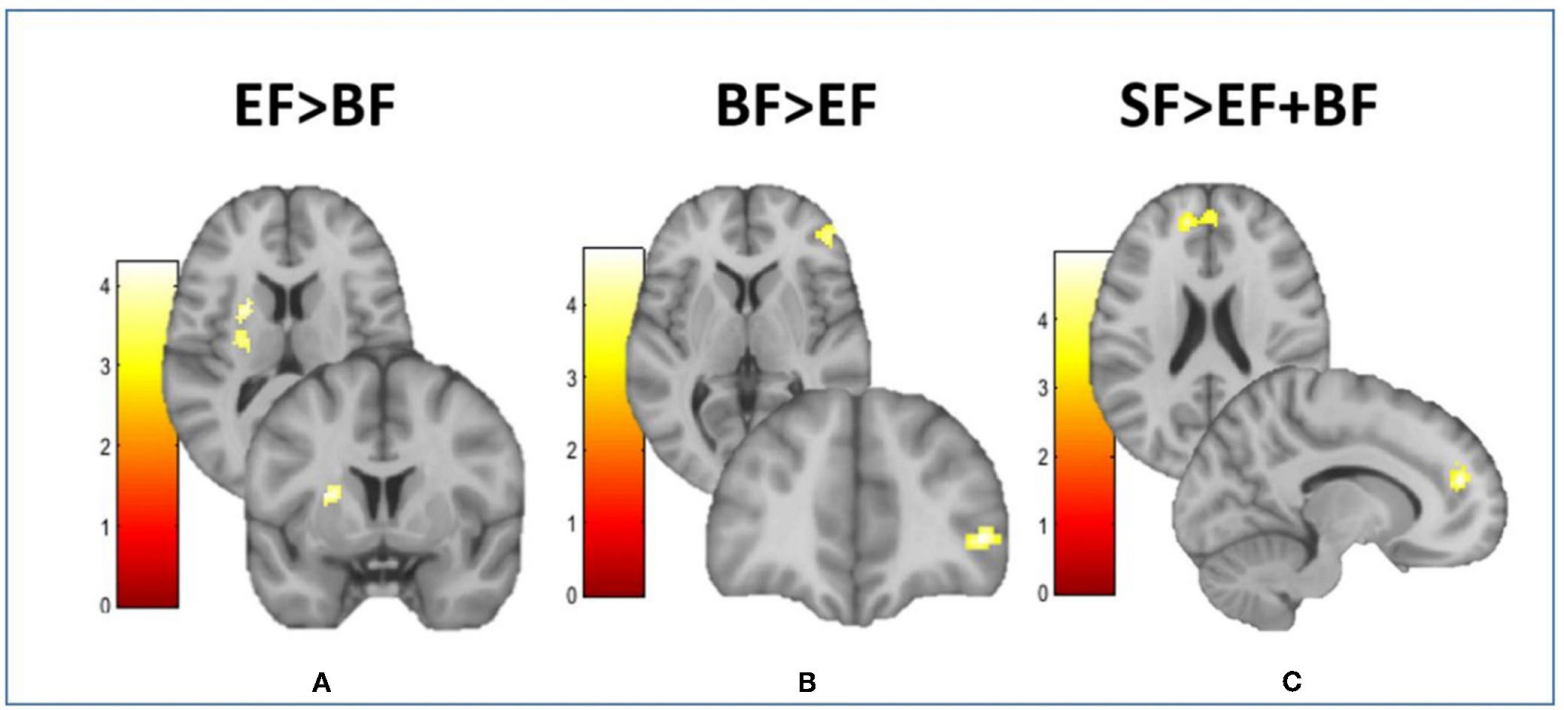
Differences between groups in the resting-state functional connectivity of the medial hypothalamus. This figure shows different brain images of the medial hypothalamus. Color bars represent the connectivity intensity value or t-value. **(A)** EF > BF: MH-putamen extending to insula; **(B)** BF > EF: MH-IFG; **(C)** SF > EF + BF: MH-dorsal ACC. ACC, anterior cingulate cortex; BF, breastfeeding; EF, experimental infant formula; IFG, inferior frontal gyrus; MH, medial hypothalamus; SF, standard infant formula. Refer to Table 2 for indicated brain regions.

Compared to the EF-fed group, BF children showed higher FC between the MH and the inferior frontal gyrus (IFG) (Figure 2B), as well as lower FC between the MH and the left putamen extending to the middle insula (Figure 2A). Moreover, those children in groups fed with EF and BF showed lower FC between the MH and the anterior cingulate cortex (ACC) in comparison with children fed with SF (Figure 2C). No differences in lateral hypothalamus FC were found between COGNIS study groups (Table 2).

**Table 2:** Study group differences in the resting-state functional connectivity of the medial hypothalamus.

**Brain region**	**H**	***x, y, z***	***t***	**CS**	**Contrast**
Putamen extending to insula	L	−22, 6, 14	4.28	116	EF > BF
IFG	R	46, 44, 2	4.73	118	BF > EF
Dorsal ACC	L	−12, 48, 18	4.96	158	SF > EF + BF

Coordinates (*x, y, z*) are given in Montreal Neurological Institute (MNI) Atlas space. ACC, anterior cingulate cortex; BF, breastfeeding; CS, cluster size; EF, experimental infant formula; H, hemisphere; IFG, inferior frontal gyrus; L, left; R, right; SF, standard infant formula; *t*, connectivity intensity value. All results herein surpassed a height threshold of *P* < 0.001 and a cluster of 110 voxels.

### Dietary intake analysis in COGNIS children up to 18 months of life

**Figure 3:**
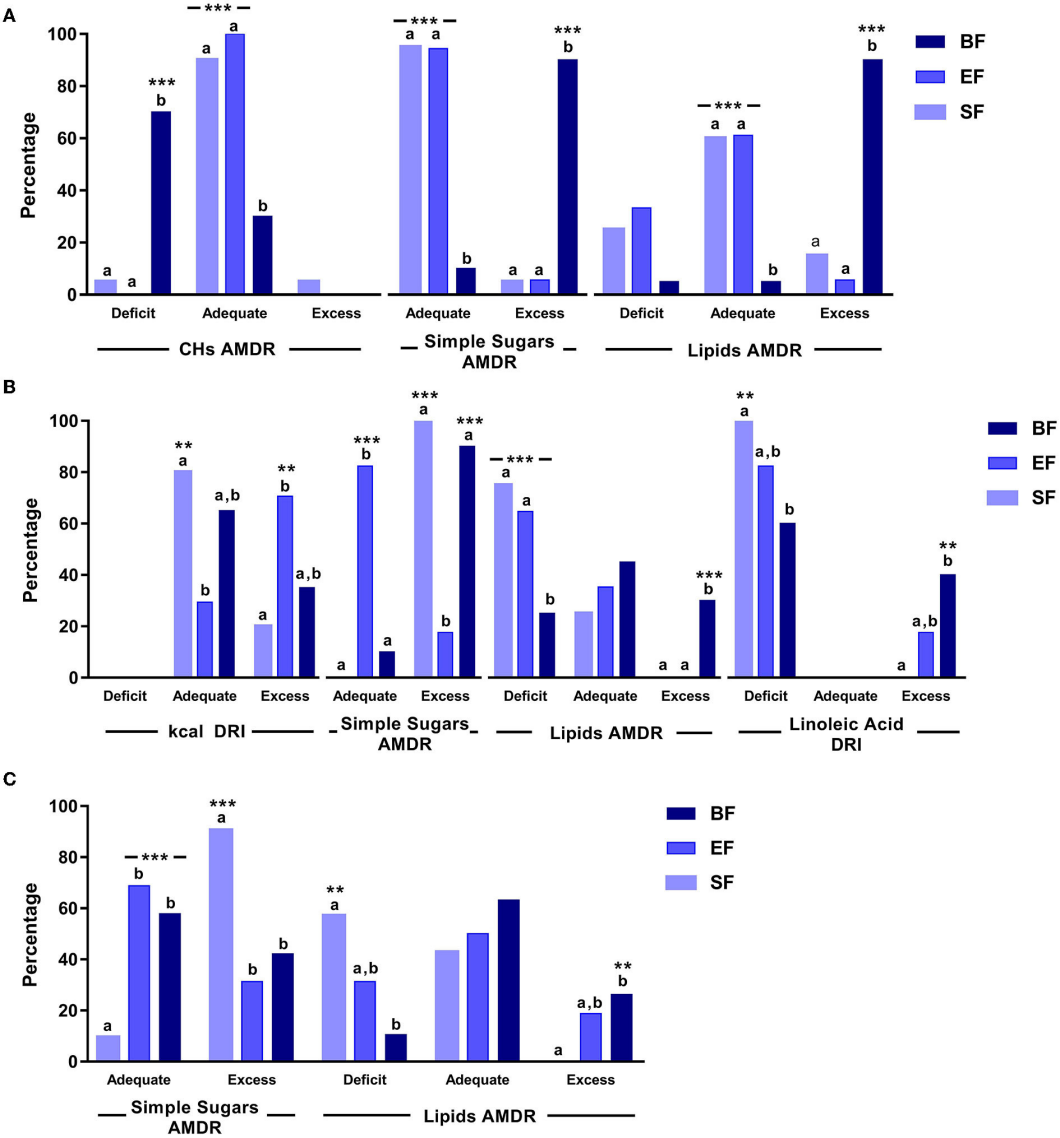
Significant differences in dietary intake in COGNIS-children up to 18 months of life. **(A)** 6 months old; **(B)** 12 months old; **(C)** 18 months old. AMDR, acceptable macronutrient distribution ranges; BF, breastfeeding; CHs, carbohydrates; DRI, dietary reference intake; EF, experimental infant formula; kcal, kilocalories; SF, standard infant formula. Simple sugars AMDR has not been determined. Values were classified as adequate or excess according to the maximal intake level, ≤ 25% of total daily energy intake (48). Values not sharing the same suffix (ab) were significantly different in the Bonferroni post-hoc test. **p < 0.01, ***p < 0.001.

Dietary intake was analyzed from 6 to 18 months of age in the three study groups to assess its adequacy to nutritional recommendations (Figure 3 and Supplementary Table 1).

At 6 months of age, groups fed with SF and EF had a more adequate CHs, simple sugars, and lipids AMDRs (%), compared to BF infants (*p* = < 0.001).

At 12 months old, the SF-fed group showed more adequate energy intake according to DRIs (g/day) compared to the EF-fed group (*p* = 0.006). However, a more adequate simple sugars AMDR was found in EF-fed infants compared to the groups fed with SF and BF (*p* = < 0.001). Finally, those infants who were breastfed showed a less lipid deficiency, considering the AMDR, compared to both formula-fed groups (*p* = 0.001), as well as a lower linolenic acid deficiency according to DRIs, compared to the SF-fed group (*p* = 0.003).

At 18 months old, a higher simple sugars AMDR was observed in SF-fed infants compared to the groups fed with BF and EF (*p* = < 0.001). Moreover, the SF-fed group also showed a lipid deficiency considering the AMDR, compared to the BF group (*p* = 0.006).

### Association between resting-state functional connectivity at 6 years old and nutrient intake during the first 18 months of life

**Figure 4:**
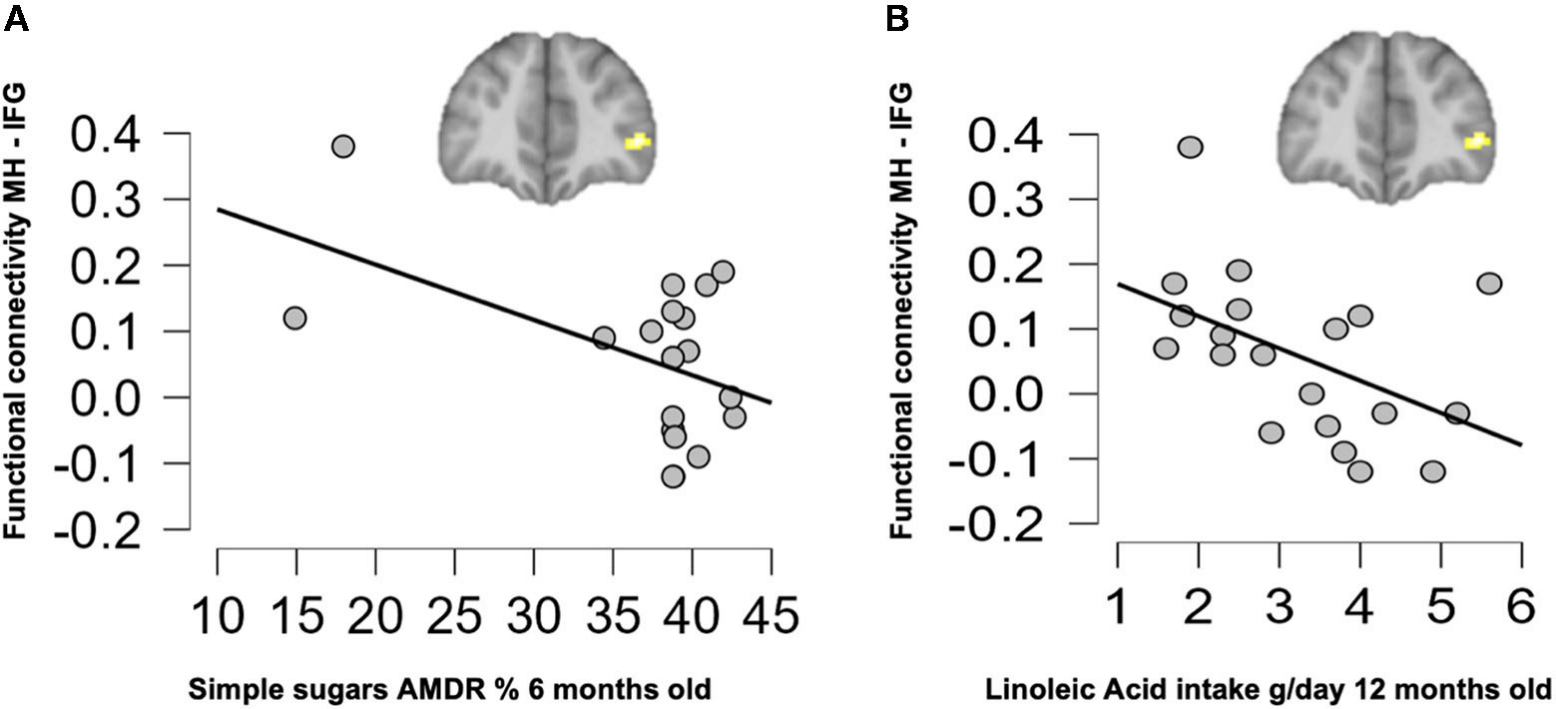
Associations between resting-state functional connectivity at 6 years old and early nutrition at 6 months **(A)** and 12 months **(B)** of age in the BF group. AMDR, acceptable macronutrient distribution ranges; BF, breastfed; IFG, inferior frontal gyrus; MH, medial hypothalamus.

Correlation analyses were performed to evaluate potential associations between resting-state FC in children at 6 years old and their nutrient intake up to 18 months of age. As shown in Figure 4A, children who were breastfed showed higher connectivity on the MH-IFG, which was associated with lower simple sugars AMDR or energy supply by simple sugars to total daily energy intake (%) at 6 months of age (*r* = −0.503; *p* = 0.047). Moreover, children in the BF group showed a negative association between this same network (MH-IFG) and linoleic acid intake (g/day) at 12 months old (*r* = −0.511; *p* = 0.043; Figure 4B). Interestingly, brain FC was not correlated with early nutrition at 18 months old in BF children. Similarly, no significant associations between early nutrition at 6, 12, and 18 months and the connectivity of hypothalamus were found in either of the infant formula-fed COGNIS groups.

### Association between mean glucose levels and resting-state functional connectivity in COGNIS children at 6 years old

Finally, we also analyzed potential associations between the resting-state FC of the hypothalamus and mean glucose levels in COGNIS children at 6 years old. However, independently of COGNIS study groups, no significant associations were found between both outcomes at this age.

## Discussion

To the best of our knowledge, this is the first study to evaluate potential long-term differences depending on the diet with a bioactive nutrient-enriched infant formula, compared to an SF or BF during the first 18 months of life, on brain function and glucose levels in healthy children at 6 years old. It is worth noting that all participants were healthy and within the normal range regarding brain function and glucose levels at 6 years of age, and no differences were found between study groups regarding the intake of macronutrients at that age. Our results showed that at 6 years old, children fed with EF and BF showed lower FC between the MH and the ventral ACC compared to SF-fed children. In addition, BF children had higher FC between the MH and the IFG and lower FC between the MH and the left putamen extending to the middle insula, compared with EF-fed children. These areas are key regions within the salience network, which is involved in processing salience stimuli, eating motivation, and hedonic-driven desire to consume food. Indeed, current higher connectivity found on the MH-IFG network in the BF group was associated with lower simple sugars AMDR at 6 months of age. Regarding linoleic acid intake at 12 months old, a negative association with the same network (MH-IFG) was found but only in the BF group.

Our results show that children fed with EF and BF had lower FC between the MH and the ventral ACC compared to SF-fed children at 6 years old. The ventral ACC is part of the salience network together with the insula and the putamen; higher FC in this network may promote the consumption of high-caloric foods (57). Therefore, it is possible that SF-fed children might be more inclined to develop unhealthier food choices compared to the children fed with EF and BF later in life, increasing the risk for obesity development. This similarity in FC between EF and BF might be due to EF was enriched with different bioactive nutrients (LC-PUFAs, MFGM components, synbiotics, gangliosides, nucleotides, and sialic acid) to narrow the nutritional gap with human breast milk. It is well known that LC-PUFAs (DHA and ARA) play a key role in brain development, contributing to the growth of neurons and synapses and the operation of neurotransmitters (58). In fact, the supplementation with fish oil (nutritional source of LC-PUFAs) during pregnancy seems to be able to shape resting-state network functioning in their children at school age (9.5–10 years old) (59). MFGM components positively influence brain development (60). Recent results from the COGNIS study suggest that the supplementation of infant formulas with MFGM components, LC-PUFAs, and synbiotics could be associated with beneficial long-term effects on neurocognitive development and brain structure at 6 years old (61). Gangliosides have important functions in neurogenesis and neural repair, being in great abundance during development in the hippocampal region of the brain, which is responsible for memory and higher cognitive function (62). Infant formulas supplemented with nucleotides have been associated with increased head growth, related to long-term cognitive function (63). In addition, sialic acid is an essential nutrient for brain development and cognition (64). At 6 years old, no significant differences in food intake were found, and children at this age are not completely autonomous regarding food decision-making, because they are not in charge of deciding what food should be bought or the daily menu of the family, which is mainly their parents' decision.

Higher connectivity between MH and IFG has been related to better food intake control and cognitive inhibition (65). Activation of the IFG is involved in suppressing the desire for food and resisting temptation, and it seems to ensure that overfeeding does not occur (66). Thus, children with a hedonically motivated eating behavior may continue to overeat later on in life; that is children with greater interest in food demonstrated weaker communication between reward and response inhibition-related regions, promoting greater enjoyment and consumption of foods, as well as eating in absence of hunger (65). In our study, BF children had higher FC between these areas, which may mean better food intake control compared to EF-fed children later in life. Indeed, shorter BF duration during infancy has been related to poorer satiety response and higher consumption in absence of hunger in adolescents (67). Thus, understanding if BF influences the development of satiety responsiveness and a less hedonically motivated eating behavior may be key, since, nowadays, food choices are characterized by abundant calorie-dense foods, and diabetes and obesity have become a worldwide health problem, affecting people of all ages (68).

In contrast, BF children also showed lower connectivity between the MH and reward-related brain areas compared to the EF-fed children group. More specifically, MH, insula, and putamen are key regions within the salience network, which is involved in the processing of salience stimuli, eating motivation, and the hedonic-driven desire to consume food (69–72). In this sense, putamen and insula are known to promote approaching behavior to foods with palatable properties, i.e., foods with high content of lipids, simple sugars, and energy (69). Shapiro et al. (65), in a study performed on non-obese children between 3 and 6 years old, found that alterations in salience and reward begin early in life and may constitute a risk for obesity development later in life *via* overeating. Since the MH is considered to be the “satiety center” of the brain, an altered FC pattern with the insula and the putamen could lead to an amplified response to food cues, promoting increased sensitivity to immediate reward and overconsumption of high-caloric foods (57). In our study, BF children presented lower FC between these areas. Thus, this may express a lesser preference for high-caloric foods or a decreased eating motivation compared to EF-fed children. This lower FC between these areas in BF children could explain the multiple health benefits attributed to breast milk, such as the high content of human milk oligosaccharides (HMOs), not present in any of the study infant formulas or any of the infant formulas commercialized when we started the COGNIS study (73–75), and its subsequent benefits over brain-gut-microbiome interactions (76). Currently, infant formulas are being supplemented with HMOs or ingredients that are functionally similar to HMOs (77), including infant formulas developed from the COGNIS study. Interestingly, a normal eating behavior has been shown to be dependent on a tightly regulated balance between intestinal and extra-intestinal homeostatic and hedonic mechanisms in the brain-gut axis. Early-life influences can prepare the gut microbiome and brain for food addiction, such as the consumption of highly palatable and high-caloric foods, shifting this balance toward hedonic eating through both central and intestinal mechanisms (76).

Remarkably, BF children showed a dual pattern of FC with brain areas. On the one hand, they showed higher FC with areas related to behavioral inhibition and, on the other hand, lower FC with reward-related areas, in comparison with EF-fed children. These results are in line with the triadic neural model in problematic eating (78), which proposes that a balanced function between prefrontal-inhibitory system, striatal-impulsive system, and insular-interoceptive system is crucial for a healthy homeostatic eating. This model also suggests that a lower prefrontal function and a hyperactive impulsive system may lead to overeating and a subsequent excess weight gain (78).

Concerning dietary intake analysis in COGNIS children, at 6 months of age, we found that groups fed with SF and EF had a more adequate CHs, simple sugars, and lipids AMDR compared to BF infants. It is worth noting that breast milk has a varying nutritional composition to adapt to infants' nutritional needs through time. Breast milk composition was not analyzed during the follow-up; thus, we estimated its composition based on a full mature breast milk composition reported in the USDA National Nutrient Database for Standard Reference (79). In addition, at 12 months old, a more adequate simple sugars AMDR was found in EF-fed infants compared to the groups fed with SF and BF, and BF infants showed less lipid deficiency according to the AMDR compared to both formula-fed groups. This could be explained because of human milk composition. It is well known that lipids are the largest source of energy in breast milk, contributing 40–55% of the total energy of breast milk (10, 80), that is why the BF group had higher lipid AMDR at 6 months of age and less lipid deficiency, considering the AMDR, at 12 months of age. In contrast, human milk contains ~7% CHs, with lactose as the main CH, and additional CH fractions, such as HMOs and fructose, contributing altogether to the higher energy supply of CHs and simple sugars to the total daily energy intake (81) compared to both infant formulas at 6 months and to EF-fed infants at 12 months of age. From 6 to 12 months of age, we observed a greater difference between BF and both formula-fed groups regarding dietary intake, because it is the period when complementary feeding is initiated, together with a gradual reduction of breast milk or infant formula intake (82). Nonetheless, at 18 months old, other dietary factors come into play, because their diet is not mainly based on breast milk or infant formula intake anymore, and they are almost completely integrated with family meals (83). Thus, we observed a closer dietary pattern between infants fed with BF and EF at this age, since we found higher simple sugars AMDR in the diet of SF-fed infants compared to the groups fed with BF and EF, and lipid deficiency considering the AMDR in SF-fed infants compared to the BF group.

Regarding associations between nutrient intake during the first 18 months of life and brain FC at 6 years old, we found that higher connectivity on the MH-IFG network was associated with lower simple sugars AMDR or, in other words, lower simple sugars energy supply to the total daily energy intake at 6 months old and only in the BF group. A higher connection between MH and IFG has been related to a more homeostatic eating behavior (65). Again, this could be explained by the presence of HMOs in breast milk, which are part of the CH components and have several health benefits (77), not present in any of the study infant formulas, as mentioned above. Therefore, it is possible that BF during the first months of life may lead to a lower simple sugars content in the diet later in life, or that lactating mothers may positively influence their offspring's diet up to 6 years, since at 6 years old, no significant differences were found regarding simple sugars intake. Then, BF influences offspring's diet during childhood, which might lead to a more advantageous FC in the MH.

Concerning linoleic acid intake at 12 months old, we found a negative association with this same network (MH-IFG) in the BF group as well. This was unexpected, considering that the BF group presented less lipid deficiency according to the AMDR compared to children from both formula-fed groups at 12 months old, as well as lower linolenic acid deficiency according to the DRI compared to the SF-fed group; furthermore, human breast milk fat provides 40–55% of the total daily energy intake in infants up to 6 months of age (80, 84), and LC-PUFAs represent about 15% of the total lipids in breast milk (84). However, studies in mice have shown that high-fat diets with high linoleic acid content (22.5% of kilocalories) seem to have an obesogenic effect, causing greater body weight gain, decreasing physical activity, and inducing insulin resistance compared to a low-fat control diet or a diet with a lower content in linoleic acid (1% of kilocalories) (85). On this matter, in another study carried out in mice fed with a high-fat diet, increased linoleic acid in the whole brain and hypothalamus was found, suggesting that this PUFA could be acting as a homeostatic suppressor in the initiation of hypothalamic inflammation triggered by saturated fatty acids, which may protect against dysfunction of hypothalamic activity (86). Therefore, a higher concentration of linoleic acid in the brain could be due to its effect as a suppressor of hypothalamic inflammation, rather than being the responsible factor for that inflammation leading to insulin resistance and other metabolic disturbances (86). Thus, linoleic acid content in the diet seems to be important to determine the effects that this PUFA could have at a brain metabolic level.

No significant correlation was found between brain FC and mean glucose levels, both measured at 6 years old, in any of the three study groups. Nonetheless, BF children had lower mean glucose levels compared to SF-fed ones. This could be explained because of many short- and long-term health benefits of breast milk. Indeed, BF has been related to a lower incidence of obesity and diabetes (type 1 and type 2) later in life, since it is inversely associated with adult risk factors for metabolic syndrome, including insulin resistance (87–89). Moreover, no correlation between brain FC and mean glucose levels could be explained because the CGM device is an innovative, although reliable, method to measure glucose levels (90), and studies on this matter are still scarce and mainly carried out in diabetic populations.

The main strength of this study is its design as a prospective, randomized, double-blind longitudinal study. To the best of our knowledge, the COGNIS study is the first study trying to analyze potential differences, depending on the type of diet during the first 18 months, on brain function (through neuroimaging examination) and mean glucose levels (through a 24-h CGM device) in healthy term infants, including a long-term follow-up, up to 6 years of age. Compared to previous studies, our nutritional intervention has added a value due to the long-term follow-up and the supplementation of the EF with several functional nutrients (LC-PUFAs, MFGM components, synbiotics, gangliosides, nucleotides, and sialic acid). Furthermore, it is well known that brain function in children is influenced by several environmental factors, such as nutrition, gender, and socioeconomic status, among others (91–94). Therefore, to obtain consistent and reliable results and conclusions, several confounding factors, previously mentioned (53–55), were taken into account in the statistical analysis.

However, this study has limitations that should be addressed as well: the drop-outs during the 6-year follow-ups, and the fact that the resting-state fMRI scanning requires that children remain still; thereby, some data were not suitable for analysis due to excessive motion. Nonetheless, it should be considered that fMRI scanning is even more difficult during the resting-state evaluation in pediatric populations. It is worth noting that not all parents who came to the 6-year follow-up visit with their children wanted them to participate in the fMRI or wear the 24-h CGM device. In this regard, the statistical power reached to detect minimum differences of 0.9 SD in brain connectivity, mean glucose values, and dietary intake between groups was 70%; thus, more studies in this field are needed to corroborate the current study findings.

## Conclusion

The results obtained in the resting-state FC evaluation by fMRI at 6 years of age may be the first to establish a possible relationship between the type of diet, breast milk, or infant formulas (standard or enriched with synbiotics, LC-PUFAs, MFGM components, gangliosides, nucleotides, and sialic acid), during the first months of life and an inclined proclivity for hedonic eating later in life, when these children become independent in their food choices. Thus, this could lead to overconsumption and a preference of eating for hedonic reasons rather than physiological ones, predisposing the development of metabolic diseases, such as T2D or obesity. As diabetes and obesity have become a worldwide health problem affecting people of all ages, and T2D is being diagnosed in children now more frequently (68), it is necessary to carry out more studies to explore the possible relationship that might exist between FC of certain brain areas and the development of unhealthy behaviors toward food. Moreover, as T2D has been associated with cognitive decline and represents an increased risk for brain diseases (95–97), it would be necessary to deepen the knowledge in this area to prevent aging brain pathologies later in life.

## Data availability statement

The raw data supporting the conclusions of this article will be made available by the authors, without undue reservation.

## Ethics statement

The studies involving human participants were reviewed and approved by Research BioEthical Committee from the University of Granada (Spain) and the BioEthical Committees for Clinical Research from San Cecilio University Clinical and University Mother-Infant Hospitals of Granada (Spain). Written informed consent to participate in this study was provided by the participants' legal guardian/next of kin.

## Author contributions

CC and AC: conceptualization. ED, AN-R, CM-P, AC, and CC: methodology. CM-P, AN-R, ED, NS-V, and AC: formal analysis. ED, AN-R, NS-V, and FH: investigation. ED, AN-R, and CM-P: writing—original draft preparation. ED, AN-R, CM-P, NS-V, JJ, RD-C, AC, JG-S, MB, and CC: writing—review and editing. AC and CC: supervision. CC: project administration. JJ, RD-C, and CC: funding acquisition. All authors have read and agreed to the published version of the manuscript.

## Funding

This project has been partially funded by Ordesa Laboratories, S.L. Contract University of Granada General Foundation, No. 3349, and SMARTFOODS (CIEN-IDI-20141206) Spanish Ministry of Economy, Industry, and Competitiveness and Contract University of Granada General Foundation, No. 4003; also partially funded by HORIZON 2020 EU DynaHEALTH Project (GA No. 633595). ED has been granted a predoctoral scholarship from Junta de Andalucía Consejería de Transformación Económica, Industria, Conocimiento y Universidades (contract code 404), Granada, Spain. NS-V has been granted a scholarship from Fundación Carolina, Madrid, Spain.

## Conflict of interest

Authors JJ and RD-C are employees of Ordesa Laboratories, S.L. The remaining authors declare that the research was conducted in the absence of any commercial or financial relationships that could be construed as a potential conflict of interest.

## Publisher's note

All claims expressed in this article are solely those of the authors and do not necessarily represent those of their affiliated organizations, or those of the publisher, the editors and the reviewers. Any product that may be evaluated in this article, or claim that may be made by its manufacturer, is not guaranteed or endorsed by the publisher.
