# Supplementary material for: Association study between hypothalamic functional connectivity, early nutrition, and glucose levels in healthy children aged 6 years: The COGNIS study follow-up
Source: Front Nutr. 2022 Oct 12;9:935740. doi: 10.3389/fnut.2022.935740 (PMC9597646; doi:10.3389/fnut.2022.935740)
Supplement: Supplementary file 1 [file Table_1.docx]

**Supplementary Table 1**. Dietary intake adequacy to nutritional recommendations in the COGNIS groups from 6 to 18 months of age

| **Nutrients** | | **6 Months** | | |  | **12 Months** | | |  | **18 Months** | | | |
| --- | --- | --- | --- | --- | --- | --- | --- | --- | --- | --- | --- | --- | --- |
|  |  | **SF** | **EF** | **BF** | ***p*¹** | **SF** | **EF** | **BF** | ***p*¹** | **SF** | **EF** | **BF** |  |
|  |  | **(n=20)** | **(n=18)** | **(n=20)** |  | **(n=20)** | **(n=17)** | **(n=20)** |  | **(n=21)** | **(n=16)** | **(n=19)** | ***p*¹** |
| kcal adequacy to DRI | Deficit | 0 (0%) | 0 (0%) | 0 (0%) | 0.348 | 0 (0%) | 0 (0%) | 0 (0%) | **0.006** | 0 (0%) | 0 (0%) | 0 (0%) | 0.802 |
|  | Adequate | 11 (55.0%) | 13 (72.2%) | 15 (75.0%) |  | 16 (80.0%)^a^ | 5 (29.4%)^b^ | 13 (65.0%)^a,b^ |  | 8 (38.1%) | 5 (31.3%) | 8 (42.1%) |  |
|  | Excess | 9 (45.0%) | 5 (27.8%) | 5 (25.0%) |  | 4 (20.0%)^a^ | 12 (70.6%)^b^ | 7 (35.0%)^a,b^ |  | 13 (61.9%) | 11 (68.8%) | 11 (57.9%) |  |
| kcal‎/kg‎/day adequacy to DRI | Deficit | 4 (20.0%) | 4 (22.2%) | 1 (5%) | 0.270 | 4 (20.0%) | 3 (17.6%) | 5 (25.0%) | 0.591 | 2 (9.5%) | 1 (6.3%) | 0 (0%) | 0.448 |
|  | Adequate | 0 (0%) | 0 (0%) | 0 (0%) |  | 3 (15.0%) | 0 (0%) | 1 (5.0%) |  | 0 (0%) | 1 (6.3%) | 2 (10.5%) |  |
|  | Excess | 16 (80.0%) | 14 (77.8%) | 19 (95.0%) |  | 13 (65.0%) | 14 (82.4%) | 14 (70.0%) |  | 19 (90.5%) | 14 (87.5%) | 17 (89.5%) |  |
| Protein AMDR | Deficit | 0 (0%) | 0 (0%) | 0 (0%) | - | 0 (0%) | 0 (0%) | 0 (0%) | - | 0 (0%) | 0 (0%) | 0 (0%) | - |
|  | Adequate | 20 (100%) | 18 (100%) | 20 (100%) |  | 20 (100%) | 17 (100%) | 20 (100%) |  | 21 (100%) | 16 (100%) | 19 (100%) |  |
|  | Excess | 0 (0%) | 0 (0%) | 0 (0%) |  | 0 (0%) | 0 (0%) | 0 (0%) |  | 0 (0%) | 0 (0%) | 0 (0%) |  |
| Grams of protein/kg/day FAO | Deficit | 0 (0%) | 0 (0%) | 0 (0%) | - | 0 (0%) | 0 (0%) | 0 (0%) | - | 0 (0%) | 0 (0%) | 0 (0%) | - |
|  | Adequate | 0 (0%) | 0 (0%) | 0 (0%) |  | 0 (0%) | 0 (0%) | 0 (0%) |  | 0 (0%) | 0 (0%) | 0 (0%) |  |
|  | Excess | 20 (100%) | 18 (100%) | 20 (100%) |  | 20 (100%) | 17 (100%) | 20 (100%) |  | 21 (100%) | 16 (100%) | 19 (100%) |  |
| CHs AMDR | Deficit | 1 (5.0)^a^ | 0 (0%)^a^ | 14 (70.0%)^b^ | **<0.001** | 1 (5.0%) | 0 (0%) | 4 (20.0%) | 0.140 | 0 (0%) | 3 (18.8%) | 6 (31.6%) | 0.110 |
|  | Adequate | 18 (90.0%)^a^ | 18 (100%)^a^ | 6 (30.0%)^b^ |  | 17 (85.0%) | 15 (88.2%) | 16 (80.0%) |  | 21 (100%) | 13 (81.3%) | 13 (68.4%) |  |
|  | Excess | 1 (5.0%) | 0 (0%) | 0 (0%) |  | 2 (10.0%) | 2 (11.8%) | 0 (0%) |  | 0 (0%) | 0 (0%) | 0 (0%) |  |
| Simple sugars AMDR* | Adequate | 19 (95.0%)^a^ | 17 (94.4%)^a^ | 2 (10.0%)^b^ | **<0.001** | 0 (0%)^a^ | 14 (82.4%)^b^ | 2 (10.0%)^a^ | **<0.001** | 2 (9.5%)^a^ | 11(68.8%)^b^ | 11 (57.9%)^b^ | **<0.001** |
|  | Excess | 1 (5.0%)^a^ | 1 (5.6%)^a^ | 18 (90.0%)^b^ |  | 20 (100%)^a^ | 3 (17.6%)^b^ | 18 (90.0%)^a^ |  | 19 (90.5%)^a^ | 5 (31.3%)^b^ | 8 (42.1%)^b^ |  |
| Lipids AMDR | Deficit | 5 (25.0%) | 6 (33.3%) | 1 (5.0%) | **<0.001** | 15 (75.0%)^a^ | 11 (64.7%)^a^ | 5 (25.0%)^b^ | **0.001** | 12 (57.1%)^a^ | 5 (31.3%)^a,b^ | 2 (10.5%)^b^ | **0.006** |
|  | Adequate | 12 (60.0%)^a^ | 11 (61.1%)^a^ | 1 (5.0%)^b^ |  | 5 (25.0%) | 6 (35.3%) | 9 (45.0%) |  | 9 (42.9%) | 8 (50.0%) | 12 (63.2%) |  |
|  | Excess | 3 (15.0%)^a^ | 1 (5.6%)^a^ | 18 (90.0%)^b^ |  | 0 (0%)^a^ | 0 (0%)^a^ | 6 (30.0%)^b^ |  | 0 (0%)^a^ | 3 (18.8%)^a,b^ | 5 (26.3%)^b^ |  |
| Linoleic acid DRI | Deficit | 11 (55.0%) | 11 (61.1%) | 17 (85.0%) | 0.069 | 19 (95.0%) | 13 (76.5%) | 17 (85.0%) | 0.318 | 20 (95.2%) | 14 (87.5%) | 19 (100%) | 0.269 |
|  | Adequate | 0 (0%) | 1 (5.6%) | 1 (5.0%) |  | 0 (0%) | 0 (0%) | 0 (0%) |  | 0 (0%) | 0 (0%) | 0 (0%) |  |
|  | Excess | 9 (45.0%) | 6 (33.3%) | 2 (10.0%) |  | 1 (5.0%) | 4 (23.5%) | 3 (15.0%) |  | 1 (4.8%) | 2 (12.5%) | 0 (0%) |  |
| Linolenic acid DRI | Deficit | 11 (55.0%) | 11 (61.1%) | 17 (85.0%) | 0.069 | 20 (100%)^a^ | 14 (82.4%)^a,b^ | 12 (60.0%)^b^ | **0.003** | 21 (100%) | 13 (81.3%) | 17 (89.5%) | 0.118 |
|  | Adequate | 0 (0%) | 1 (5.6%) | 1 (5.0%) |  | 0 (0%) | 0 (0%) | 0 (0%) |  | 0 (0%) | 0 (0%) | 0 (0%) |  |
|  | Excess | 9 (45.0%) | 6 (33.3%) | 2 (10.0%) |  | 0 (0%)^a^ | 3 (17.6%)^a,b^ | 8 (40.0%)^b^ |  | 0 (0%) | 3 (18.8%) | 2 (10.5%) |  |
| n6 PUFAs AMDR | Deficit | 15 (75.0%) | 13 (72.2%) | 11 (55.0%) | 0.348 | 19 (95.0%) | 15 (88.2%) | 19 (95.0%) | 0.669 | 21 (100%) | 15 (93.8%) | 18 (94.7%) | 0.528 |
|  | Adequate | 5 (25.0%) | 5 (27.8%) | 9 (45.0%) |  | 1 (5.0%) | 2 (11.8%) | 1 (5.0%) |  | 0 (0%) | 1 (6.3%) | 1 (5.3%) |  |
|  | Excess | 0 (0%) | 0 (0%) | 0 (0%) |  | 0 (0%) | 0 (0%) | 0 (0%) |  | 0 (0%) | 0 (0%) | 0 (0%) |  |
| n3 PUFAs AMDR | Deficit | 19 (95.0%) | 17 (94.4%) | 20 (100%) | 0.756 | 19 (95.0%) | 16 (94.1%) | 20 (100%) | 0.746 | 21 (100%) | 15 (93.8%) | 19 (100%) | 0.275 |
|  | Adequate | 1 (5.0%) | 1 (5.6%) | 0 (0%) |  | 1 (5.0%) | 1 (5.9%) | 0 (0%) |  | 0 (0%) | 1 (6.3%) | 0 (0%) |  |
|  | Excess | 0 (0%) | 0 (0%) | 0 (0%) |  | 0 (0%) | 0 (0%) | 0 (0%) |  | 0 (0%) | 0 (0%) | 0 (0%) |  |

Data are presented as n (%) for categorical data. ^1^ *p*-values are comparisons between COGNIS-groups. Chi-Square test was carried out for categorical variables. *Fisher’s exact test for categorical variables. Values which do not share the same suffix (ab) are significantly different in a Bonferroni post-hoc test. Bold: *p*-value <0.05*.* AMDR: Acceptable Macronutrient Distribution Ranges; BF: breastfed infants; CHs: carbohydrates; DRI: dietary reference intake; EF: experimental infant formula; FAO: the Food and Agriculture Organization of the United Nations (46); kcal: kilocalories; SF: standard infant formula; n3 PUFAs: omega 3 polyunsaturated fatty acids; n6 PUFAs: omega 6 polyunsaturated fatty acids. *Simple sugars AMDR has not been determined. The values were classified as adequate or excess according to the maximal intake level, ≤25% of total daily energy intake (48).

Supplementary Table 2. Nutritional composition of Standard (SF) and Experimental (EF) Infant Formulas used in the COGNIS study.

|  | **Standard Infant Formula (SF)** | | **Experimental Infant Formula (EF)** | |
| --- | --- | --- | --- | --- |
|  | **Initiation** | **Follow-on** | **Initiation** | **Follow-on** |
|  | 100 ml (13.5%) | 100 ml (14.5%) | 100 ml (13.5%) | 100 ml (14.5%) |
| **Energy** (kcal/kJ) | 69/288 | 70/294 | 68/285 | 69/290 |
| **Proteins** (g) | 1.35 | 1.8 | 1.35 | 1.8 |
| Casein/whey (%) | 40/60 | 50/50 | 40/60 | 50/50 |
| **Carbohydrates** (g) | 7.97 | 8.5 | 7.56 | 8.1 |
| Lactose (g) | 7.17 | 7.2 | 6.82 | 7.3 |
| Maltodextrin (g) | 0.8 | 1.3 | 0.7 | 0.8 |
| **Fat** (g) | 3.5 | 3.2 | 3.5 | 3.2 |
| LA (mg) | 579 | 517 | 569 | 517 |
| α-ALA (mg) | 49 | 45 | 49 | 45 |
| ARA (mg) | - | - | 15.8 | 10.2 |
| DHA (mg) | - | - | 11.2 | 10.2 |
| **Gangliosides** (mg/L) | 1.5 | 1.5 | 9 | 9 |
| **Sialic acid** (mg/L) | 82 | 80 | 105 | 105 |
| **MFGM-10** (wt/wt) | - | - | 10% | 10% |
| **Nucleotides** (mg) | - | - | 2.92 | 2.94 |
| Cytidine-5'-Monophosphate (mg) | - | - | 1.09 | 1.12 |
| Uridine-5'-Monophosphate (mg) | - | - | 0.88 | 0.9 |
| Adenosine-5'-Monophosphate (mg) | - | - | 0.41 | 0.41 |
| Guanosine-5'-Monophosphate (mg) | - | - | 0.27 | 0.26 |
| Inosine-5'-Monophosphate (mg) | - | - | 0.27 | 0.26 |
| **Prebiotics** |  |  |  |  |
| *FOS: Inulin 1:1* | - | - | 0.4 | 0.4 |
| **Probiotics** |  |  |  |  |
| *Bifidobacterium L.infantis* CECT7210 (*Bifidobacterium infantis*IM1) | - | - | 1x10^7^ cfu/g | 1x10^7^ cfu/g |
| *Lactobacillus rhamnosus* LCS-742 | - | - | 1x10^7^ cfu/g | 1x10^7^ cfu/g |

Initiation formula: Up to 6 months old. Follow-on formula: from 6 to 18 months old. ARA: Arachidonic acid; cfu: colony forming unit; DHA: Docosahexaenoic acid; FOS: Fructooligosaccharides; LA: Linoleic acid; MFGM: Milk Fat Globule Membrane; α-ALA: α-Linolenic acid. Power diluted 13.5% (13.5 g infant formula made up to 100 ml with water); power diluted 14.5% (14.5 g infant formula made up to 100 ml with water).

**Supplementary Methodology**

**Dietary Reference Intakes (DRIs)** (48) **at 6, 12 and 18 months of age**

At 6 months, kilocalories (kcal) adequacy to DRI was classified as deficit <494.45 kcal/day, adequate 494.45-805.95 kcal/day or excess >805.95 kcal/day; kcal‎/kg‎/day adequacy to DRI as deficit <82.00 kcal/kg/day, adequate 82.00-84.00 kcal/kg/day or excess >84 kcal/kg/day; grams of protein/kg/day according to Food and Agriculture Organization of the United Nations (FAO) recommendations (46) as deficit <0.98 grams of protein/kg/day, adequate 0.98-1.14 grams of protein/kg/day, or excess >1.14 grams of protein/kg/day; linoleic acid DRI as deficit <4.4 g/day, adequate 4.4 g/day or excess >4.4 g/day.

At 12 months, kcal adequacy to DRI was classified as deficit <576.15 kcal/day, adequate 576.15-967.75 kcal/day or excess >967.75 kcal/day; kcal‎/kg‎/day adequacy to DRI as deficit <78.00 kcal/kg/day, adequate 78.00-82.00 kcal/kg/day or excess >82 kcal/kg/day; grams of protein/kg/day according to FAO recommendations as deficit <0.95 grams of protein/kg/day, adequate 0.95-1.14 grams of protein/kg/day or excess >1.14 grams of protein/kg/day; linoleic acid DRI as deficit <4.6 g/day, adequate 4.6 g/day or excess >4.6 g/day.

At 18 months, kcal adequacy to DRI was classified as deficit <672.05 kcal/day, adequate 672.05-1045.85 kcal/day or excess >1045.85 kcal/day; kcal‎/kg‎/day adequacy to DRI as deficit <80.00 kcal/kg/day, adequate 80.00-83.00 kcal/kg/day or excess >83 kcal/kg/day; grams of protein/kg/day according to FAO recommendations as deficit <0.85 grams of protein/kg/day, adequate 0.85-1.03 grams of protein/kg/day, or excess >1.03 grams of protein/kg/day; linoleic acid DRI as deficit <7.0 g/day, adequate 7.0 g/day or excess >7.0 g/day; linolenic acid DRI as deficit <0.7 g/day, adequate 0.7 g/day or excess > 0.7 g/day.

At 6 and 12 months, linolenic acid DRI was classified as deficit <0.5 g/day, adequate 0.5 g/day or excess > 0.5 g/day. At 6, 12 and 18 months, protein Acceptable Macronutrient Distribution Range (AMDR) (48) was classified as deficit <5% of energy, adequate 5-20% of energy or excess >20% of energy; carbohydrates AMDR as deficit <45% of energy, adequate 45-65% of energy or excess >65% of energy; AMDR for simple sugars has not been determined. These values correspond to the maximal intake level, considering as adequate ≤25% of total daily energy intake or excess >25% of total daily energy intake; lipids AMDR as deficit <30% of energy, adequate 30-40% of energy or excess >40% of energy; omega 6 polyunsaturated fatty acids AMDR as deficit <5% of energy, adequate 5-10% of energy or excess >10% of energy; omega 3 polyunsaturated fatty acids AMDR as deficit <0.60% of energy, adequate 0.60-1.20% of energy or >1.20% of energy.
